# Role of Machine Learning and Artificial Intelligence in the Diagnosis and Treatment of Refractive Errors for Enhanced Eye Care: A Systematic Review

**DOI:** 10.7759/cureus.57706

**Published:** 2024-04-06

**Authors:** Taghreed A Alnahedh, Mohammed Taha

**Affiliations:** 1 Optometry, King Abdullah International Medical Research Center (KAIMRC), National Guard Health Affairs, Riyadh, SAU; 2 Academic Affairs, King Saud Bin Abdulaziz University for Health Sciences College of Medicine, Riyadh, SAU; 3 Ophthalmology, King Saud Bin Abdulaziz University for Health Sciences College of Medicine, Riyadh, SAU

**Keywords:** a systematic review, optometry, refractive error, machine learning, artificial intelligence

## Abstract

A significant contributor to blindness and visual impairment globally is uncorrected refractive error. To plan effective interventions, eye care professionals must promptly identify people at a high risk of acquiring myopia, and monitor disease progress. Artificial intelligence (AI) and machine learning (ML) have enormous potential to improve diagnosis and treatment. This systematic review explores the current state of ML and AI applications in the diagnoses and treatment of refractory errors in optometry. A systematic review and meta-analysis of studies evaluating the diagnostic performance of AI-based tools in PubMed was conducted according to the Preferred Reporting Items for Systematic Reviews and Meta-Analyses (PRISMA) guidelines. To find relevant studies on the use of ML or AI in the diagnosis or treatment of refractive errors in optometry, a thorough search was conducted in various electronic databases such as PubMed, Google Scholar, and Web of Science. The search was limited to studies published between January 2015 and December 2022. The search terms used were "refractive errors," "myopia," "optometry," "machine learning," "ophthalmology," and "artificial intelligence." A total of nine studies met the inclusion criteria and were included in the final analysis. ML is increasingly being utilized for automating clinical data processing as AI technology progresses, making the formerly labor-intensive work possible. AI models that primarily use a neural network demonstrated exceptional efficiency and performance in the analysis of vast medical data, rivaling board-certified, healthcare professionals. Several studies showed that ML models could support diagnosis and clinical decision-making. Moreover, an ML algorithm predicted future refraction values in patients with myopia. AI and ML models have great potential to improve the diagnosis and treatment of refractive errors in optometry.

## Introduction and background

Refractive errors are common vision problems that occur when the eye cannot focus light correctly on the retina. These errors include myopia (nearsightedness), hyperopia (farsightedness), astigmatism, and presbyopia. Myopia, a common refractive error, has become a major public health concern due to its association with adverse changes in the eye tissue [1]. Even mild myopia increases the risk of eye problems (like retinal blur, accommodation problem). High myopia and pathological myopia (PM) significantly raise the risk of irreversible vision loss, including glaucoma, retinal detachment, myopic macular degeneration (MMD), and macular choroidal neovascularization [2].

A significant contributor to blindness and visual impairment globally is the uncorrected refractive error. Refractive error-related blindness affected three to four million people worldwide as of 2020, whereas 140-175 million people had vision impairment [3]. These figures are expected to increase significantly in the future because of factors including population aging (which can cause presbyopia) and lifestyle choices (which can lead to myopia) [4,5]. Indeed, myopia and presbyopia are predicted to cause yearly global productivity losses of US$ 244 billion and US$ 25.4 billion, respectively, which illustrates the huge economic impact of refractive errors [6,7].

The World Health Organization (WHO) has emphasized the need to include eye care in universal health coverage (UHC) to address the inequities in access to and delivery of these services among populations [8]. As part of this initiative, the WHO is developing a collection of eye care interventions (Package of Eye Care Interventions, or PECI) that nations may use to organize their national health services, packages, and policies and to plan how to allocate resources and implement eye care interventions [9]. A key component of the PECI is that, whenever possible, the interventions chosen should be supported by substantial evidence.

The ability of eye care professionals to plan effective interventions depends on the prompt identification of people at a high risk of acquiring myopia and the regular, repetitive monitoring of its development and associated difficulties [2]. The growing weight of myopia, however, might be too great for the current healthcare systems to handle [4].

Machine learning (ML), a subset of artificial intelligence (AI), primarily uses computer system programming to carry out tasks or forecast outcomes [10]. AI and ML have enormous potential in clinical settings to aid diagnosis and treatment, including in the field of optometry [11]. ML and AI technologies offer new opportunities to improve the accuracy and efficiency of refractive error detection and treatment. For instance, deep machine learning may be trained on copious amounts of imaging and clinical data to support clinical ophthalmology [12]. ML can also identify biological markers that can be difficult to identify even by human experts, such as retinal discoveries from fundus images linked to cardiovascular risk [13].

Worldwide, there is a deficiency of qualified myopia specialists, making it challenging for general eye care professionals like optometrists or general ophthalmologists to diagnose myopic maculopathy. Additionally, it is inefficient to continuously monitor every myopic patient, in terms of both time and money. Likewise, the COVID-19 epidemic has highlighted the value of remote testing and monitoring even more. Fortunately, the combination of telemedicine and AI technology can help close this gap. This systematic review aimed to identify and assess available data on the use of AI and ML in myopia diagnosis and treatment. The evidence generated through this review will inform decisions around the utility of using AI and ML in the diagnosis and treatment of myopia in optometry globally.

## Review

Methods

Study Selection

This study explores the application of AI and ML in diagnosing and treating myopia in optometry. The study used the Preferred Reporting Items for Systematic Review and Meta-Analyses (PRISMA) approach. MEDLINE, Web of Science, PubMed, and Google Scholar databases were searched for the selection of relevant articles.

Eligibility Criteria

Inclusion criteria:* *This study explored the application of AI and ML in diagnosing and treating refractive errors in optometry. Studies that reported the use of ML or AI in diagnosing or treating myopia in optometry were included. The search was limited to studies published between January 2015 and December 2022. Studies published in the English language, studies that investigated inherited retinal diseases or their subtypes and used at least one of the AI and ML techniques were included in the review.

Exclusion criteria:* *Studies primarily focused on diseases other than refractive diseases and published before January 2015 and after December 2022 were excluded from the study. Likewise, studies that were not published in the English language were excluded.

Data Extraction

Characteristics of the eligible studies were extracted using the standard data extraction sheet. Data extraction was conducted by two independent reviewers who scanned the retrieved articles. The main outcome of the study was the use of AI and ML in myopia diagnosis, and treatments were assessed across most of the studies. A combination of Medical Subject Heading (MeSH) keywords and text words were identified. The search terms used were "refractive errors," "myopia," "optometry," "machine learning," "ophthalmology," and "artificial intelligence." Reference lists of included studies were subsequently reviewed to identify additional studies that met the predefined inclusion criteria. Next, we integrated these key ideas utilizing combinations (AND, OR) that were pertinent to the study issue. Additionally, we utilized the same root word truncation (*) to find other study publications. We also imposed publication period limitations and search limits or filters on the language (English).

Results

**Figure 1:**
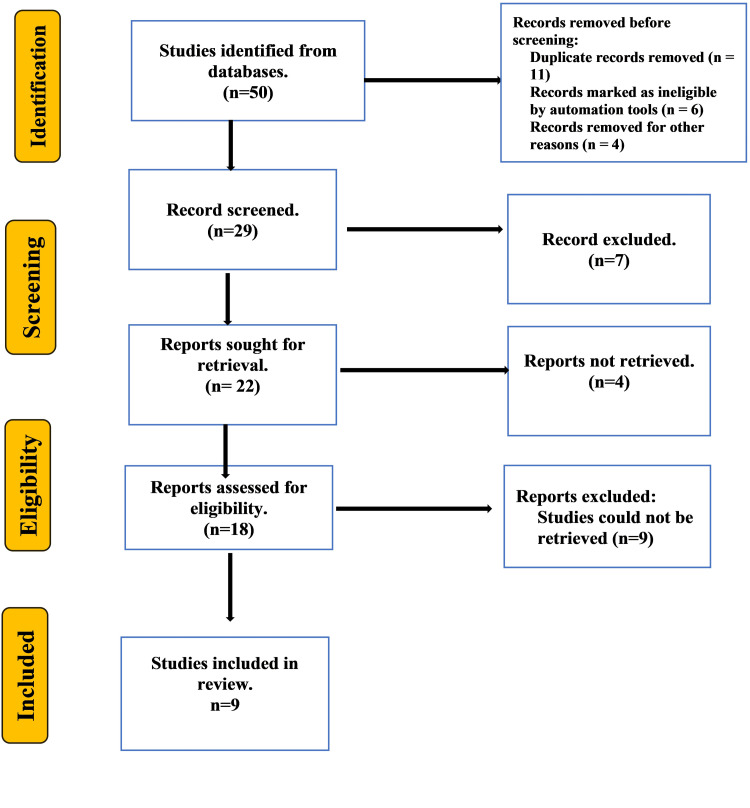
Preferred Reporting Items for Systematic Reviews and Meta-Analyses (PRISMA) flow chart of the included studies

The initial screening yielded 50 studies. The detailed search strategy for the review is explained in detail in Figure 1. After removing duplicate entries and eliminating records that did not meet inclusion and exclusion criteria, the systematic search yielded nine studies for review and analysis. A summary of included studies is presented in Table 1. The findings were evaluated to identify themes and concepts, which are discussed.

**Table 1:** Summary of included studies

Author	Title	Method	Findings	Reported limitation(s)
Achiron et al. [14]	Predicting refractive surgery outcome: machine learning approach with big data	Decision forest for the prediction of laser refractive surgery outcome	Machine learning models can be used for clinical decision-making and may lead to better individual risk assessment.	Single parameter used; single-center study.
Yoo et al. [15]	Explainable machine learning approach as a tool to understand factors used to select the refractive surgery technique on the expert level	A multiclass XGBoost model for selecting refractive surgery option	Machine learning models could support clinical decision-making and may lead to better individual risk assessment.	Single-center; no absolute criteria for corneal surgery; interclinician variability not determined.
Malik et al. [16]	Data driven approach for eye disease classification with machine learning	General framework to use machine learning models for diagnosis	Machine learning models achieved high accuracy and outperformed the traditional diagnostic methods used by medical experts.	General model, not evaluated for specific disease; small sample size.
Lin et al. [17]	Prediction of myopia development among Chinese school-aged children using refraction data from electronic medical records: a retrospective, multicentre machine learning study	Machine learning algorithm	Machine learning algorithm achieved clinically acceptable prediction of the actual refraction values at future time points.	An algorithm that is created using a training set could overfit.
Fageeri et al. [18]	Eye refractive error classification using machine learning techniques.	Decision tree (J48)	The machine learning algorithm, J48 classifier outperforms both Naïve Bayesian as well as SVM.	Not reported
Scanzera et al. [19]	Optometrist’s perspectives of artificial intelligence in eye care	Survey	Optometrists are optimistic about the use of AI in eye care.	Single-survey pilot study; with online surveys, there is a possibility that a single participant filled multiple forms.
Anton et al. [20]	Comprehensive review on the use of artificial intelligence in ophthalmology and future research directions	Surveys	AI potentially increases the patient's access to screening/clinical diagnosis and decreases healthcare costs	Not reported
Ting et al. [12]	Development and validation of a deep learning system for diabetic retinopathy and related eye diseases using retinal images from multiethnic populations with diabetes	Deep learning systems	The study found that a deep learning system (DLS) had high accuracy in identifying diabetic retinopathy, possible glaucoma, and age-related macular degeneration in multiethnic populations with diabetes, indicating its potential to improve vision outcomes.	Training not developed based on retinal specialists; DLS analyses each retinal image using many levels of representation, omitting to display the actual diabetic retinopathy lesions; diabetic macular edema not identified in all cases.
Fan et al. [21]	Machine learning algorithm improves accuracy of ortho-K lens fitting in vision shaping treatment	Machine learning models	The machine learning model can provide practitioners with an efficient method for estimating the alignment curve curvatures of vision shaping treatment lenses and reducing the probability of cross-infection originating from trial lenses.	Not reported

ML and AI Models for Predicting Refractive Surgery Outcomes

The reviewed literature suggested that ML models have the potential to enhance various aspects of eye care, such as predicting refractive surgery outcomes, diagnosing myopia, and improving lens fitting accuracy. Specifically, two studies by Achiron et al. and Yoo et al. utilized decision forest and multiclass XGBoost models to develop predictive models [14,15]. The results indicate that these models have high accuracy in predicting the outcomes of laser refractive surgery and selecting the most suitable refractive surgery option, respectively, thereby supporting clinical decision-making and enhancing individual risk assessment.

ML and AI Models for Individual Risk Assessment

Two studies specifically focused on predicting refractive surgery outcomes using ML models. Achiron et al. and Yoo et al. both showed that these models can be used to support clinical decision-making and lead to better individual risk utilized a decision forest model to predict the outcomes of laser refractive surgery [14,15]. At the same time, Yoo et al. used a multiclass XGBoost model to select the most appropriate refractive surgery option [15]. Both studies achieved high accuracy in their predictions, indicating the potential of ML in reducing the risk of complications and improving patient satisfaction.

These findings highlight the potential of ML models to enhance the quality of eye care and personalized treatment for individual patients. By accurately predicting outcomes and selecting the most appropriate treatment options, these models can reduce the risk of complications and improve patient satisfaction, leading to better overall outcomes in eye care.

ML and AI Models for Predicting Refractive Disease Classification

The systematic review revealed that ML models have the potential to enhance accuracy in the diagnosis and treatment of myopia. Malik et al. demonstrated the potential of ML models in achieving high accuracy in eye disease classification [16]. The study developed a general framework for ML models and achieved high accuracy in classifying various types of eye diseases. The findings suggest that ML models can outperform traditional diagnostic methods used by medical experts in diagnosing eye diseases. This has significant implications for improving patient outcomes and reducing the risk of misdiagnosis. By utilizing ML models, healthcare professionals can achieve faster and more accurate diagnoses, leading to earlier interventions and improved patient care. The findings of this systematic review further highlight the potential of machine learning in enhancing various aspects of eye care, including myopia surgery, disease diagnosis, and lens fitting accuracy.

Deep convolutional neural networks (CNNs) and other deep neural networks have been successfully used to develop AI systems for automated computer-assisted detection (CAD), leveraging large clinical databases [22]. The role of CNNs in the context of eye diseases, particularly diabetic retinopathy (DR), has been highlighted in several studies [12,23,24]. These studies have shown the effectiveness of deep learning models, specifically CNNs, in detecting and grading the severity of DR lesions.

Myopia has been proposed as a non-modifiable factor that can reduce the incidence and severity of DR. On the other hand, refractive error and DR stage measures were recorded over a longer period of time in a study involving a sizable cohort of type 1 diabetes patients. The results of the studies demonstrated that in this group, myopia did not reduce the probability of developing DR in comparison to emmetropia. Furthermore, the results imply that hyperopia is a separate risk factor for proliferative DR (PDR) and the advancement of two- and three-step DR [25]. Moreover, a study introduced a two-stage deep CNN model that focused on both lesion detection and grading the severity of DR, demonstrating promising results in accurately identifying DR lesions and categorizing them based on their severity [23]. By leveraging the capabilities of CNNs, this approach can potentially aid clinicians in diagnosing and monitoring DR more efficiently. Another study proposed a CNN model that not only detects DR lesions but also incorporates heatmaps into the model to enhance explainability. Explainability, also known as interpretability, describes the degree to which the ML output can be explained to or understood by a human being. By utilizing heatmaps, the model provides visual representations of the regions within the retinal images that contribute most to the detection and severity grading of DR. This added explainability can be valuable in clinical practice, as it helps clinicians understand and interpret the model's predictions, leading to increased trust and confidence in its results [26].

ML and AI Models for Predicting Myopia

The systematic review supported the potential for ML models to improve eye care outcomes. Lin et al. demonstrated the effectiveness of ML models in predicting myopia development among school-aged children using refraction data from electronic medical records [17]. The study found that the ML algorithm achieved clinically acceptable prediction of actual refraction values at future time points, indicating its potential in detecting myopia earlier and enabling timely intervention to prevent its progression.

Moreover, Fageeri et al. showed the potential of ML models in eye refractive error classification [18]. The study compared the performance of different classifiers, including Naïve Bayesian, J48, a development of the ID3 algorithm that is a conventional algorithm, and support vector machines (SVMs), in classifying eye refractive errors. The J48 classifier was found to outperform other classifiers, indicating its potential to improve the accuracy of eye refractive error classification. These findings suggest that ML models can play a crucial role in improving eye care outcomes and enhancing the accuracy of various eye-related diagnoses.

ML and AI Models for Screening Myopia

The survey conducted by Scanzera et al. indicated that optometrists are optimistic about the use of AI in myopia care [19]. The study suggests that AI has the potential to improve efficiency and quality of care and increases access to screening/clinical diagnosis while reducing healthcare costs. Fan et al. utilized a machine learning algorithm to improve the accuracy of ortho-K lens fitting in vision shaping treatment (VST), which is a non-surgical alternative for myopia. The study showed that ML models can improve the accuracy of ortho-K lens fitting in VST [21]. The study suggested that the ML model can provide practitioners with an efficient method for estimating the alignment curve (AC) curvatures of VST lenses and reducing the probability of cross-infection originating from trial lenses. The rising prevalence of myopia is linked to an earlier onset of the condition, which increases the likelihood of advancing to high myopia. This tendency is alarming, especially because high myopia, an aggravated form of the illness, has been related to an increased susceptibility to serious ocular consequences such as myopic maculopathy, retinal detachment, and glaucoma [27]. Myopic maculopathy consequences can have a significant influence on an individual's quality of life, resulting in lifelong vision loss and disability [28].

Discussion

The systematic review revealed that ML and AI models have the potential to enhance various aspects of eye care: accuracy in the diagnosis, diagnosing myopia, predicting refractive surgery outcomes, improving lens fitting accuracy and treatment of myopia. By utilizing these models, healthcare professionals can achieve faster and more accurate diagnoses, leading to earlier intervention and improved patient care. The reviewed literature suggested that ML models have the potential to enhance the quality of personalized treatment for individual patients. By accurately predicting outcomes and selecting the most appropriate treatment options, these models can reduce the risk of complications and improve patient satisfaction, leading to better overall outcomes in eye care.

Conventional techniques of measuring myopia are time-consuming and labor-intensive, and require expensive instruments in the hands of trained professionals. Individuals with communication issues, such as small children, the elderly, and those with language impediments, may find it challenging to comply during such examinations [29]. Furthermore, in resource-limited situations, the paucity of medical equipment and specialists makes precisely assessing refractive errors difficult, resulting in lost treatment chances and irreparable vision loss. As a result, there is an urgent need for affordable, high-quality refraction services that are widely accepted by the public.

ML is increasingly being utilized for automating clinical data processing as AI technology progresses, making formerly labor-intensive work less time-consuming [30]. AI models that use ML models as their foundation have demonstrated exceptional efficiency and performance in the analysis of vast medical data, rivaling board-certified healthcare professionals [31-33]. AI-powered diagnosis software has been used successfully in screening for diabetic retinopathy and glaucoma [34,35]. ML models can also help in selecting the appropriate surgery option for patients. The field of ophthalmology has seen significant advancements in recent years with the introduction of big data and ML techniques. These techniques have the potential to revolutionize the diagnosis and treatment of myopia, glaucoma, and other eye diseases. Our review of recent studies highlights the potential benefits of using ML in ophthalmology.

One of the key benefits of ML is its ability to assist in clinical decision-making. Studies have shown that ML-based decision forests can support clinical decision-making in refractive surgeries, leading to better individual risk assessment and improved patient outcomes [14]. ML models have also been developed to recommend an optimal laser refractive surgery option for patients based on expert clinical decisions and ophthalmic measurements, outperforming traditional diagnostic methods used by medical experts [16]. CNN-based models have shown promise in accurately detecting and grading DR lesions, making them a valuable tool for clinicians, while incorporating explainability features such as heatmaps enhances the interpretability of the models, further increasing their utility in clinical practice.

In addition, ML can accurately diagnose eye diseases such as glaucoma and dry eye syndrome. For instance, a study found that ML algorithms could accurately classify glaucoma based on retinal nerve fiber layer thickness and visual field examination through feature evaluation, achieving high accuracy, sensitivity, specificity, and area under the curve [36]. Another study demonstrated that ML techniques could be used to produce an objective, repeatable, and automatic diagnosis for dry eye syndrome [37]. Furthermore, ML approaches have been applied to improve the prediction of myopia prognosis in children. A study utilized real-world clinical data to develop an algorithm that could predict the onset of high myopia among Chinese school-aged children up to 10 years in the future, achieving clinically acceptable accuracy [17]. This has significant implications for clinical practice, health policy-making, and individualized interventions for myopia control. Fundus photography is primarily utilized for capturing images of the retina and diagnosing conditions related to the posterior eye segment. Fundus photography is integral for detecting retinal conditions, in addition to a comprehensive eye examination, necessitating the use of distinct tools for the accurate assessment of myopia, keratometry, and anterior chamber depth [38].

Finally, optometrists have demonstrated a willingness to incorporate AI into their practice to increase their efficiency and improve the patient experience [19]. The combination of AI and ML algorithms using both structured and unstructured data has the potential to increase patient access to screening and clinical diagnosis while decreasing healthcare costs, particularly in communities facing financial shortages [20].

Challenges and Future Direction

While there has been a significant advancement in the use of ML and AI in eye care, several issues still need to be resolved. Data security and privacy are among the key issues, as the usage of patient data in these models poses privacy concerns. Therefore, it is necessary to employ strong data protection procedures to guarantee the confidentiality of sensitive data.

The trustworthiness and comprehensibility of ML and AI models is another issue. Many models' "black-box" character makes it challenging for clinicians to comprehend and trust the decision-making process. Explainable AI models may help to alleviate these concerns, including local interpretable model-agnostic explanations (LIME) and Shapley additive explanations (SHAP) [39]. These methods attempt to identify the areas and characteristics in the source images that have the greatest influence on the model's predictions and offer insights into the model's decision-making process.

Another issue that needs to be addressed is integration with currently used workflows and systems in the field of optometry. The widespread acceptance and successful application of ML and AI technologies in clinical practice depend on their seamless integration. The success of ML and AI models also depends on having access to high-quality data. Effective training of these models requires the availability of a wide variety of representative data. This obstacle can be removed by standardizing data collecting and sharing amongst institutions, which will also make it easier for these technologies to be used widely in optometry. In conclusion, despite the enormous potential that ML and AI have demonstrated for the diagnosis and treatment of refractive defects, overcoming issues with data privacy, explainability, integration, and data quality is crucial for their general adoption and successful use in clinical practice.

Limitations

There are a few limitations to this systematic review that should be noted. First, the number of studies included in the review was relatively small, limiting the finding's generalizability. The studies were also conducted in different populations, using different methods and machine learning models, making it difficult to directly compare the results. Another limitation is the possibility of publication bias, where studies with significant results are more likely to be published than those with non-significant results. This could potentially skew the overall findings of the review. Lastly, there may be limitations in the quality and bias of the individual studies included in the review. While efforts were made to select studies with robust methodology and appropriate sample sizes, there may still be biases or confounding factors that were not fully accounted for in the studies. Overall, while the findings of this systematic review highlight the potential of ML models in various aspects of eye care, it is important to consider these limitations and to conduct further research to fully understand the benefits and limitations of these models in clinical practice.

## Conclusions

In conclusion, the current evidence supports the importance of ML and AI in optometry. This systematic review demonstrates the potential of ML and AI models in improving various aspects of eye care, particularly in supporting clinical decision-making, diagnosing eye diseases, predicting disease progression, and myopia treatment. The mass acceptance of these technologies can be aided via the development of explainable AI models. While further research is needed to validate these findings, the potential benefits of ML in ophthalmology are clear, and ML and AI will likely continue to play an increasingly important role in the field in the future.
